# Comparative Transcriptome Analysis Reveals the Intensive Early Stage Responses of Host Cells to SARS-CoV-2 Infection

**DOI:** 10.3389/fmicb.2020.593857

**Published:** 2020-11-25

**Authors:** Jiya Sun, Fei Ye, Aiping Wu, Ren Yang, Mei Pan, Jie Sheng, Wenjie Zhu, Longfei Mao, Ming Wang, Zanxian Xia, Baoying Huang, Wenjie Tan, Taijiao Jiang

**Affiliations:** ^1^Center for Systems Medicine, Institute of Basic Medical Sciences, Chinese Academy of Medical Sciences & Peking Union Medical College, Beijing, China; ^2^Suzhou Institute of Systems Medicine, Suzhou, China; ^3^Key Laboratory of Medical Virology, National Health and Family Planning Commission, National Institute for Viral Disease Control and Prevention, Chinese Center for Disease Control and Prevention, Beijing, China; ^4^Department of Otolaryngology, Head and Neck Surgery, Beijing TongRen Hospital, Capital Medical University, Beijing, China; ^5^Hunan Key Laboratory of Animal Models for Human Diseases, Hunan Key Laboratory of Medical Genetics & Center for Medical Genetics, School of Life Sciences, Central South University, Changsha, China

**Keywords:** SARS-CoV-2, host early response, tmprss2, Calu-3 cell, time-series transcriptome

## Abstract

Severe acute respiratory syndrome coronavirus 2 (SARS-CoV-2) has caused a widespread outbreak of highly pathogenic coronavirus disease 2019 (COVID-19). It is therefore important and timely to characterize interactions between the virus and host cell at the molecular level to understand its disease pathogenesis. To gain insights, we performed high-throughput sequencing that generated time-series data simultaneously for bioinformatics analysis of virus genomes and host transcriptomes implicated in SARS-CoV-2 infection. Our analysis results showed that the rapid growth of the virus was accompanied by an early intensive response of host genes. We also systematically compared the molecular footprints of the host cells in response to SARS-CoV-2, SARS-CoV, and Middle East respiratory syndrome coronavirus (MERS-CoV). Upon infection, SARS-CoV-2 induced hundreds of up-regulated host genes hallmarked by a significant cytokine production, followed by virus-specific host antiviral responses. While the cytokine and antiviral responses triggered by SARS-CoV and MERS-CoV were only observed during the late stage of infection, the host antiviral responses during the SARS-CoV-2 infection were gradually enhanced lagging behind the production of cytokine. The early rapid host responses were potentially attributed to the high efficiency of SARS-CoV-2 entry into host cells, underscored by evidence of a remarkably up-regulated gene expression of TPRMSS2 soon after infection. Taken together, our findings provide novel molecular insights into the mechanisms underlying the infectivity and pathogenicity of SARS-CoV-2.

## Introduction

Coronavirus disease 2019 (COVID-19) triggered by the severe acute respiratory syndrome coronavirus 2 (SARS-CoV-2) is currently affecting global health. The SARS-CoV-2 is the third highly pathogenic coronavirus following SARS-CoV and Middle East respiratory syndrome coronavirus (MERS-CoV) that cause severe accurate respiratory symptoms in humans. Since December 2019, this virus has caused more than 80 thousand COVID-19 cases in China. Nowadays, the number of infections in countries outside China is growing rapidly. The most remarkable feature of the SARS-CoV-2 incidences and epidemiology is its great capacity for human-to-human transmission (Li et al., 2020). Clinically, the majority of COVID-19 patients have mild and moderate symptoms, and the elderly appear to have severe symptoms (Guan et al., 2020). Based on the analysis of China data, the COVID-19 case-fatality rate was estimated at around 4.0% (3,341 deaths over 82,249 confirmed cases of SARS-CoV-2 infection) (Verity et al., 2020), lower than those of SARS and MERS (Song et al., 2019). However, due to the large-scale infected population, the SARS-CoV-2 has already caused more than 1,081,868 deaths as of October 14, 2020 (WHO, 2020), sowing great social panic around the world.

While recent efforts have been focused on transcriptome analysis of host responses to SARS-CoV-2 infection at a certain time point in certain cell lines (Blanco-Melo et al., 2020; Xiong et al., 2020), the transcriptional dynamics of host responses to the virus infection has remained largely unexplored. Generally, once the virus enters the cell, the host innate immune responses, such as the interferon-mediated antiviral responses and cytokine production, play a pivotal role in suppressing the virus replication, which, if inadequate, might contribute to the viral pathogenesis. This hypothesis has been supported by our previous study, which has shown that the high pathogenicity of avian influenza virus is associated with abnormal coordination between interferon-mediated antiviral responses and cytokine production in host cells (Sun et al., 2019). Similar to both SARS-CoV and MERS-CoV, which induce the overactivation of cytokines (Channappanavar and Perlman, 2017; Song et al., 2019), increased cytokine levels are also observed in patients hospitalized with COVID-19 (Huang et al., 2020). Transcriptome analysis of *in vitro* host cells shows that SARS-CoV and MERS-CoV elicit distinct responses to the expression of the host genes (Josset et al., 2013). Until now, the time-series gene expression profiling of the host response to SARS-CoV-2 remains unknown and thus is urgently needed uncovering its pathogenesis.

In this study, we used the SARS-CoV-2 strain isolated from patients (Zhu et al., 2020) to infect *in vitro* Calu-3 cells and performed RNA sequencing to determine the time-series transcriptome profiling data of the host. We established the host response patterns for SARS-CoV-2 by comprehensive analysis of the transcriptomic profiles from SARS-CoV-2, SARS-CoV, and MERS-CoV. These results provide profound new insights into the pathogenesis and progression of the COVID-19 disease caused by SARS-CoV-2, illuminating new strategies for the prevention and control of SARS-CoV-2 transmission and eventually leading to a cure of the COVID-19 disease.

## Materials and Methods

### Cells and Virus

Calu-3 human airway epithelial cells (ATCC, HTB-55) were cultured in minimum essential media (MEM) (HyClone) supplemented with 10% fetal bovine serum (FBS), 1% MEM non-essential amino acid (NEAA), and 100 U/ml penicillin–streptomycin solution (Gibco, Grand Island, NY, United States) at 37°C in a humidified atmosphere of 5% (v/v) CO_2_. Vero cells (ATCC, CCL-81) were cultured at 37°C in Dulbecco’s modified Eagle’s medium (DMEM) (Gibco) supplemented with 10% FBS (Gibco) in an atmosphere with 5% CO_2_. SARS-CoV-2 strain BetaCoV/Wuhan/IVDC-HB-01/2019 (C-Tan-HB01, GISAID accession no. EPI_ISL_402119) was isolated from a human patient (Zhu et al., 2020). Viruses were harvested, and viral titrations were performed in Vero cells using plaque assay.

### Calu-3 Cell Infections and RNA Isolation

All experiments involving infectious virus were performed in approved biosafety level 3 (BSL) laboratories at the National Institute for Viral Disease Control and Prevention, China CDC. Cells were washed with MEM and inoculated with viruses at a multiplicity of infection (MOI) of 5 or mock-diluted in MEM for 2 h at 37°C. Following inoculation, cells were washed three times with MEM, and fresh medium was added to signify 0 h. Triplicate samples of mock-infected and virus-infected Calu-3 cells were harvested at different times between 0 and 24 h post-infection (hpi). Calu-3 cells were cultured for RNA isolation using TRIzol reagent (Invitrogen, United States) following the manufacturer’s protocol.

### Virus Titration in Cell Culture Supernatants

The 50% tissue culture infectious dose (TCID50) per ml was determined for SARS-CoV-2 in Vero cells. In short, Vero cells (2 × 10^5^ cells/well) were seeded into 96-well plates and infected with cell culture supernatants in a dilution ratio of 1:10. After adsorption for 1 h at 37°C, the cell-free medium was removed, cells were washed with DMEM, and then fresh medium (DMEM containing 2% FBS) was added to cells. After 72 h, cells were observed to evaluate cytopathic effect. The TCID50 values were calculated using the Reed–Muench equation.

### Library Construction and Sequencing

A total amount of 50 ng RNA per sample was used as input material for the total RNA library construction and host rRNA removal according to the instructions of the Trio RNA-Seq kit (Nugen, 0506-32). Total RNA libraries were sequenced on the Illumina Novaseq using the 2 × 150 bp paired-end read setting.

### Data Analysis

Raw reads were filtered to obtain clean data by Trimmomatic (v0.35) (with parameters “ILLUMINACLIP:adapter.fa:2:30:10 HEADCROP:10 LEADING:3 TRAILING:3 SLIDINGWINDOW:4:15 MINLEN:36”) (Bolger et al., 2014). The cleaned data were mapped to the human GRCh38 reference genome using STAR aligner (v2.7.2a) (Dobin et al., 2013). The htseq-count command was used to count reads mapped to each gene (Anders et al., 2015). The R package DESeq2 was applied to further identify differentially expressed genes (DEGs) [false discovery rate (FDR) < 0.05, | log2FC| ≥ 1] (Love et al., 2014). The unmapped reads against the entire human genome were further aligned to the reference genome of SARS-CoV-2 (EPI_ISL_402119). Virus genome annotation was based on our previous work (Wu et al., 2020). Gene Ontology (GO) enrichment analysis was performed by Fisher’s exact test with 19,932 human protein-coding genes as a background in R. The GO terms of enrichment analysis were generated from the “gene2go” file^1^, in which redundant GO terms were further trimmed by the following criteria. First, if one pair of parent and child GO terms had the same genes, only the parent GO term was kept. Second, if one pair of siblings GO terms had the same genes, one sibling GO term was omitted. Third, only GO terms with high quality evidence codes (“TAS,” “IDA,” “IMP,” “IGI,” “IPI,” “IEP,” “ISS”) were used. GO term relationships were extracted from the “go-basic.obo” file^2^. For analysis of microarray data of SARS-CoV and MERS-CoV, normalization and identification of DEGs (FDR < 0.05, | log2FC| ≥ 1) were conducted using the R package limma (Ritchie et al., 2015) by following the steps in our previous study (Sun et al., 2019).

### Quantitative Real-Time PCR

The same RNA samples of RNA-Seq were used for quantitative real-time PCR (qRT-PCR). qRT-PCR analysis was conducted on the 7500 Fast (Life Technology) by using HiScript II One Step qRT-PCR SYBR Green Kit (Vazyme) according to the manufacturer’s instructions. Among housekeeping genes that were downloaded from this paper (Eisenberg and Levanon, 2013), 10 genes showed no significant differential expression at any time point during SARS-CoV-2 infection, in which ATF4 gene was used as reference gene of qRT-PCR. The gene primers were as follows: ATF4 (F: CTCCGGGACAGATTGGATGTT, R: GGCTGCTTATTAGTCTCCTGGAC), TMPRSS2 (F: CAAGTG CTCCAACTCTGGGAT, R: AACACACCGATTCTCGTCCTC), ACE2 (F: ACAGTCCACACTTGCCCAAAT, R: GAGAGCACT GAAGACCCATT), DPP4 (F: TACAAAAGTGACATGCCTCA GTT, R: TGTGTAGAGTATAGAGGGGCAGA), DDX58 (F: TG CGAATCAGATCCCAGTGTA, R: TGCCTGTAACTCTATACC CATGT), IFNB1 (F: GCTTGGATTCCTACAAAGAAGCA, R: ATAGATGGTCAATGCGGCGTC), IFNAR2 (F: TCATGGT GTATATCAGCCTCGT, R: AGTTGGTACAATGGAGTGGT TTT), IL6 (F: ACTCACCTCTTCAGAACGAATTG, R: CCA TCTTTGGAAGGTTCAGGTTG), IL1B (F: ATGATGGCTTA TTACAGTGGCAA, R: GTCGGAGATTCGTAGCTGGA), and TNF (F: GAGGCCAAGCCCTGGTATG, R: CGGGCCGAT TGATCTCAGC).

## Results

### Transcriptome Profiling of Virus–Host Interactions Following SARS-CoV-2 Infection

**FIGURE 1:**
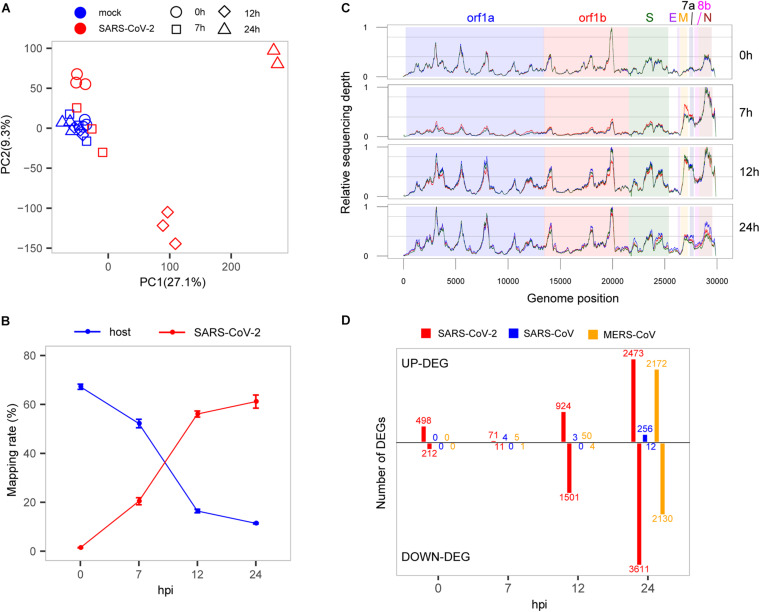
Interaction between SARS-CoV-2 and cell host. **(A)** PCA of mock- and SARS-CoV-2 infected samples. **(B)** Read mapping rate to the host or virus genomes. **(C)** Activity distribution of virus genome over times. The *y*-axis is the relative sequencing depth that is normalized by (x-min)/(max-min) across the whole genome positions. Each line represents one biological replicate. **(D)** The numbers of DEGs at each time point among the three viruses. Only protein-coding genes were counted for SARS-CoV-2.

We carried out time-course experiments to identify dynamic changes in transcripts in response to SARS-CoV-2 based on the infected and mock-infected groups across four time points (0, 7, 12, and 24 hpi), in which three biologically independent replicates for each treatment group were used for constructing cDNA libraries. The Calu-3 human airway epithelial cell line, a model of human respiratory disease (Love et al., 2014), was used as the host cell of SARS-CoV-2, subjected to the same MOI and host cell used in the previous analyses of SARS-CoV and MERS-CoV infections. The previous datasets of SARS-CoV and MERS-CoV used a sub-population of Calu-3 (Calu-3 2B4 sorted by ACE2 antibody (Josset et al., 2013)) with better infection ability than Calu-3. In order to allow similar cell entry of SARS-CoV-2, Calu-3 cells were incubated with viruses for 2 h before infection timing. After the total RNA isolation and sequencing, we obtained the host transcriptomes, as well as the genomes and transcripts of viruses. The high-throughput sequencing resulted in an average of 49 million paired-end reads per sample, and the sequencing quality was high with a mean mapping rate of unique reads at approximately 72% among mock samples (Supplementary Table S1). The quality control of all samples was assessed by the principal component analysis (PCA) based on normalized counts from DESeq2, which indicated that high quality was achieved given that the majority of samples were well clustered except for only one sample from the infection group at 24 hpi that was removed before further analysis (Figure 1A).

### Rapid Growth of SARS-CoV-2 Accompanied by Dynamic Changes of Host Genes

To evaluate the growth rate of SARS-CoV-2, we calculated the RNA level of the virus represented by unique reads mapping rates at different time points. Our results showed that in general, the virus reads increased sharply from 1.4 to 61.2%, whereas reads mapped to the host genome dropped rapidly from 67.2 to 11.4% (Figure 1B), suggesting a rapid replication of the virus within 24 h. From the results, at the earliest time point (0 hpi), the virus produced high-levels of viral genome RNA as evidenced by relatively even coverage depth across the whole genome (Figure 1C). Interestingly, we found that there was a significant active transcription of the 3′ end of SARS-CoV-2 at 7 hpi, especially for the M, 6, 7a, 7b, 8b, and N genes (Figure 1C), which could play important roles in the antagonism with host immune response (Lim et al., 2016; Fung and Liu, 2019). After that, the relatively even depth distribution of reads along viral genome was again observed at panels of 12 and 24 hpi. These time-dependent patterns of virus replication and transcription were most likely to play critical roles in the pathology of SARS-CoV-2.

To elucidate the global changes of host gene expression along with virus growth, we identified the overall up- and down-regulated DEGs during SARS-CoV-2 infection (Figure 1D and Supplementary Table S2). As shown in Figure 1D, during the early stage of infection before 7 hpi, there were many more up-regulated genes than down-regulated genes (498 vs. 212 at 0 hpi, 71 vs. 11 at 7 hpi), and soon after, the number of down-regulated genes significantly exceeds that of up-regulated genes (924 vs. 1,501 at 12 hpi, 2,473 vs. 3,611 at 24 hpi). Although the numbers of DEGs were influenced by cutoffs of fold change, there were still many significantly dysregulated genes at the early stage of SARS-CoV-2 infection by using different cutoffs (Supplementary Figure S1). Most importantly, most of the DEGs at 0 hpi were suppressed at 7 hpi, which simultaneously occurred with active transcription of the 3′ end of SARS-CoV-2 genome, demonstrating the critical role of the 3′ end in antagonizing host immune response. The suppression of host responses was not likely due to sequencing bias because the three samples from the infected group at 7 hpi were clustered with mock samples (Figure 1A). Interestingly, there seems to be some correlation between the decrease in the levels of the host transcriptome (compared with the total RNA level of SARS-CoV-2) and the relative number of up-regulated genes (compared with down-regulated genes) (Supplementary Figure S2). This may indicate the complex molecular behavior of the host cell in response to the virus infection.

### Comparison of Host Transcriptome Responses to SARS-CoV-2, SARS-CoV, and MERS-CoV

To investigate specific host responses during SARS-CoV-2 infection, we performed a comparative transcriptome analysis by integrating two public host transcriptomes of SARS-CoV (GSE33267) (Sims et al., 2013) and MERS-CoV (GSE45042) (Josset et al., 2013) infected in the same cell line with the same MOI. Overall, a huge divergence was presented in time-specific DEG patterns among SARS-CoV-2, SARS-CoV, and MERS-CoV (Figure 1D). For SARS-CoV-2, 710 DEGs (498 up-regulated and 212 down-regulated) were immediately induced at the very early stage (0 hpi), and many more DEGs were gradually observed at the late stages (12 and 24 hpi). In contrast, SARS-CoV- and MERS-CoV-infected cells exhibited far fewer DEGs (0, 4, and 3 for SARS-CoV and 0, 6, and 54 for MERS-CoV) at the early stages (0, 7, and 12 hpi). However, more DEGs were clearly detected at 24 hpi during SARS-CoV and especially MERS-CoV infection (268 and 4,302, respectively). These distinct DEG patterns indicated that SARS-CoV-2 actually induced earlier host responses than SARS-CoV and MERS-CoV.

**FIGURE 2:**
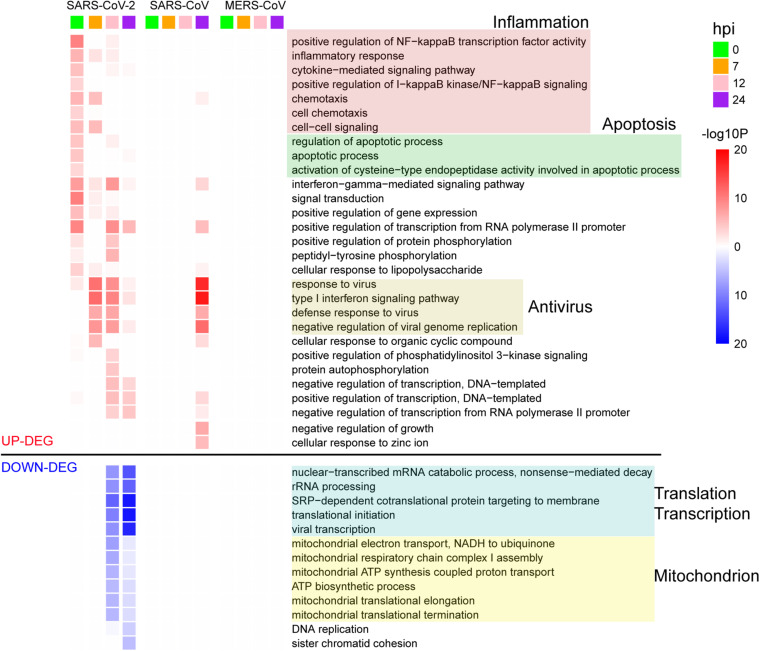
GO enrichment analysis of DEGs for the three viruses. The GO BP terms with enrichment FDR < 0.001 are shown.

To further delineate differential perturbation of pathways among three viruses, we conducted GO-enrichment analysis based on their respective DEGs. Overall, substantially enriched pathways, such as inflammation, apoptosis, antiviral response, transcription, translation, and mitochondrion-related pathways, were detected at various time points during SARS-CoV-2 infection (Figure 2). At 0 hpi, the up-regulated DEGs were mostly enriched in the inflammation-related pathways including the nuclear factor kappa B (NF-kB) signaling and cytokine-mediated signaling pathways, suggesting that SARS-CoV-2 could induce inflammatory responses at the very early stage of infection. At the same time, SARS-CoV-2 also triggered the cellular apoptosis signaling pathway, implying that early onset cell death happened along with inflammation response. Beginning at 7 hpi, our results showed a significant enrichment in antivirus response-related pathways until 24 hpi (Figure 2). At the late stages (12 and 24 hpi), down-regulated DEGs were exclusively enriched in fundamental host pathways responsible for RNA processing and transcription, protein translation, and mitochondrial activity (Figure 2). Moreover, based on gene expression levels at 0 hpi and other time points, all of the DEGs during SARS-CoV-2 infection were divided into 10 different gene patterns, in which early and late dysregulated DEGs corresponded to distinct biological functions, respectively (Supplementary Figure S3 and Supplementary Table S3). Different from SARS-CoV-2, at the late stage of SARS-CoV infection (24 hpi), the highly enriched genes were identified to be involved in antivirus-related pathways, whereas no significantly enriched pathways were found for MERS-CoV infection despite many DEGs existing at 24 hpi (Figure 2). Taken together, the above results indicated that the etiology mechanism of SARS-CoV-2 was different from that of SARS-CoV and MERS-CoV as implicated by the overall differential patterns of the host response against infection.

### Quantification of the Capacity for Host Antiviral Immunity and Cytokine Production for SARS-CoV-2, SARS-CoV, and MERS-CoV Infections

**FIGURE 3:**
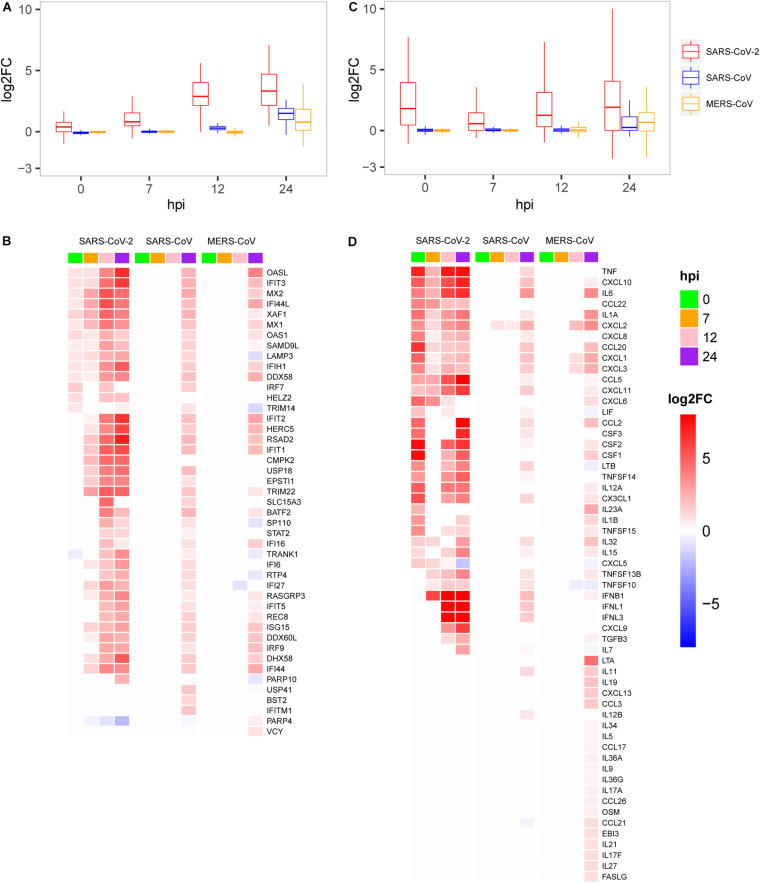
Expression patterns of the host antiviral-related genes and cytokines. **(A)** Quantification of host antiviral capacity. **(B)** Expression patterns of the host antiviral-related genes. **(C)** Quantification of the host cytokine genes. **(D)** Expression patterns of the host cytokine genes.

As mentioned above, SRAR-CoV-2 induced specific patterns of host antiviral and inflammation responses compared with SARS-CoV and MERS-CoV. To quantify host antiviral capacity and inflammation responses during infection of the three viruses, two sets of genes were used as their indicators. First, we used a set of 45 early induced genes in interferon-α treated Calu-3 cell (Sun et al., 2019) as antiviral indicators to quantify the level of host antiviral capacity against SARS-CoV-2, SARS-CoV, and MERS-CoV infections. Our analysis showed that the antiviral capacity of the host against SARS-CoV-2 was gradually increased over the time course of infection (Figure 3A). In contrast, the host antiviral capacities against both SARS-CoV and MERS-CoV were nearly zero at least during the initial stages of infection (between 0 and 12 hpi), followed by a marginal increase at 24 hpi. The antiviral capacity in SARS-CoV- and especially MERS-CoV-infected cells was much lower than that in SARS-CoV-2-infected cells, which might underpin the disparity in mortality between the three viruses. Despite the observation of the potent early induced host antiviral activity during SARS-CoV-2 infection as compared with SARS-CoV and MERS-CoV infection, our results clearly showed that most of the genes (25/45) were significantly induced among infections of the three viruses (Figure 3B). In addition, a list of the virus-specific antiviral-related genes was identified, including PARP10 (Yu et al., 2011) and CMPK2 (El-Diwany et al., 2018) for SARS-CoV-2, BST2 (Perez-Caballero et al., 2009), ITITM1, and USP41 for SARS, and PARP4 (Daugherty et al., 2014) for MERS-CoV (Figure 3B).

Second, we further used a set of 113 human cytokines to quantify host inflammation responses between three viruses. The 113 cytokines from the CytoReg database were often cited by various publications and play a primary role in the immune system (Carrasco Pro et al., 2018). Our results showed that, for SARS-CoV-2, the level of cytokine production was highly induced at 0 hpi, decreased at 7 hpi, and then slowly recovered thereafter (Figure 3C). Relatively high levels of cytokine expression only occurred at 24 hpi for SARS-CoV and MERS-CoV. Our analysis also provided evidence that SARS-CoV-2 had more cytokines in common with SARS-CoV than with MERS-CoV (Figure 3D). Unlike the other two viruses, MERS-CoV specifically induced the expression of dozens of cytokines, such as LTA, IL19, CXCL13, and CCL3, at 24 hpi, which were not observed in the case of the other two viruses. Interestingly, among the 28 up-regulated cytokines at the very early stage (0 hpi) during SARS-CoV-2 infection, eight cytokines including IL-6 (IL6), IL-1b (IL1B), IL-8 (CXCL8), G-CSF (CSF3), GM-CSF (CSF2), IP10 (CXCL10), MCP1 (CCL2), and TNF were reported to exhibit substantially elevated serum levels (Huang et al., 2020; Qin et al., 2020; Xu et al., 2020), which indicated that early induction of cytokines played critical roles in the pathology of SARS-CoV-2. While most of the eight cytokines were moderately up-regulated at the late stage during SARS-CoV and MERS-CoV infections, up-regulation was not observed at the early stage. Collectively, SARS-CoV-2 induced distinct patterns of host antiviral response and cytokine production.

### Regulation of Key Genes From Cell Entry to Type-I Interferon Production

**FIGURE 4:**
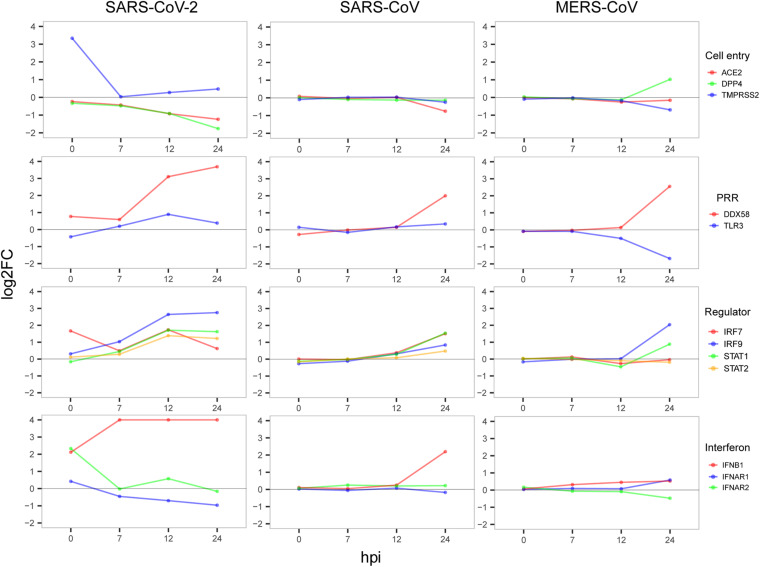
Dynamic expression of four types of important genes. The value of the *y*-axis was restricted to have a maximum of 4 to show notable gene expression changes.

Next, to gain possible explanations for the distinct patterns in host antiviral capacity and cytokine production during SARS-CoV-2 infection, dynamic expressions of four types of key genes were evaluated, including virus receptors for cell entry, pathogen recognition receptors (PRRs) for an innate immune startup, and regulator genes for induction of antiviral-related genes and interferon production (Figure 4). For the three cell entry-related genes [ACE2 as the receptor of SARS-CoV and SARS-CoV-2 (Li et al., 2003; Hoffmann et al., 2020), DPP4 as the receptor of MERS-CoV (Raj et al., 2013), and protease TMPRSS2 for S protein priming of SARS-CoV-2 (Carrasco Pro et al., 2018)], we observed the dramatic changes in TMPRSS2 expression with very early induction during SARS-CoV-2 infection and the slightly down-regulated expression of ACE2 in cells infected with SARS-CoV-2 and SARS-CoV, whereas DPP4 was more up-regulated in MERS-CoV (Figure 4). For the two PRRs, DDX58 is a canonical RIG-I-like receptor for RNA virus recognition (Kell and Gale, 2015), and TLR3 is a Toll-like receptor playing important roles in initiating a protective innate immune response to highly pathogenic coronavirus infections (Totura et al., 2015). We observed that all three viruses had a notably up-regulated expression of DDX58, whereas only MERS-CoV had a suppressed TLR3 at 24 hpi (Figure 4), which is consistent with the fact that decreased expression of TLR3 contributes to the pathology of highly pathogenic coronavirus infections (Totura et al., 2015). Among the four regulator genes, IRF7 is responsible for the expression of most IFN-α subtypes and the type I IFN amplification loop (Lazear et al., 2013), and IRF9, STAT1, and STAT2 form the ISGF3 complex that binds to interferon-stimulated response elements and thereby induces the expression of interferon-stimulated genes (Schreiber and Piehler, 2015). As expected, gradual up-regulation of the four primary regulator genes was observed for all three viruses (Figure 4). At last, we found an apparent difference in the expression of IFNB1 between SARS-CoV-2, SARS-CoV, and MERS-CoV, indicating that IFNB1 likely accounted for the observed variations of the host antiviral capacities among three viruses (Figure 4). Taken together, early induction of TMPRSS2 and gradual increased expression level of IFNB1 were likely responsible for the distinct host immune response patterns of SARS-CoV-2 infection.

### qRT-PCR Validation of Gene Expression Levels

**FIGURE 5:**
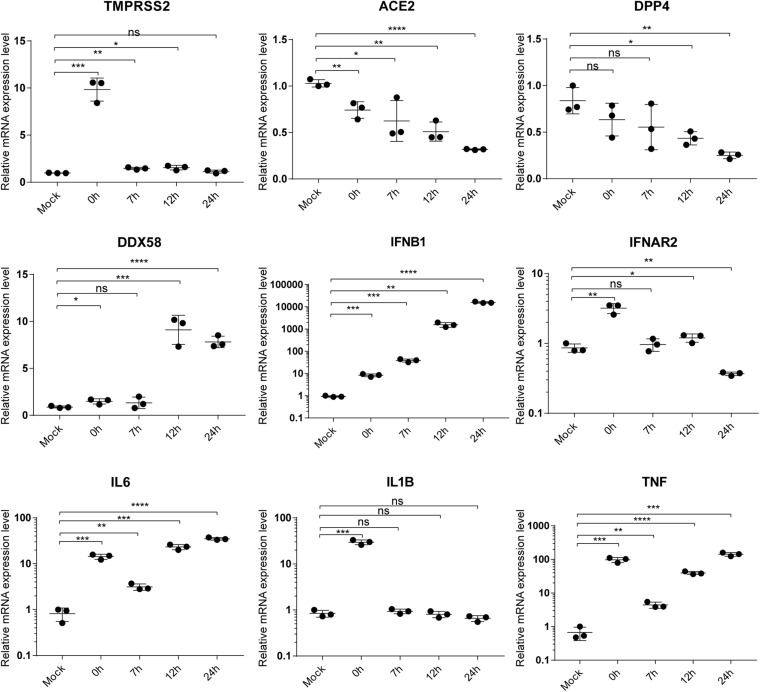
qRT-PCR validation of gene expression. The label “mock” indicates mock samples at 0 hpi. The labels from 0 to 24 h represent infected samples at indicated time points. Statistical significance is tested using *t*-test. “*” denotes significant difference and “ns” for no significance. The error bars represent mean ± SD. *p < 0.05, **p < 0.01, ***p < 0.001 and ****p < 0.0001.

To validate the accuracy of RNA-Seq gene expression levels, qRT-PCR experiments were performed for nine genes including TMPRSS2, ACE2, DPP4, DDX58, IFNB1, IFNAR2, IL6, IL1B, and TNF. Here, a housekeeping gene ATF4 without significant differential expression at any time point during SARS-CoV-2 infection was used as reference gene of qRT-PCR. As shown in Figure 5, expression levels of the nine genes exhibited high consistencies with those of RNA-Seq (Figures 3, 4), which supported that gene expression quantification from RNA-Seq was reliable.

## Conclusion

Using time-series profiling of the virus genome and host transcriptome at the same time during SARS-CoV-2 infection coupled with comparative transcriptome analysis, we found that, compared with SARS-CoV and MERS-CoV, SARS-CoV-2 induces strong host cell responses at the very early stage of infection that not only favor its high infectivity to host cells but also restrict its pathogenesis.

## Discussion

Here, we sequenced the transcriptomes of SARS-CoV-2- and virus-infected host cells simultaneously during the early stages of infection, providing a robust reference dataset to speculate the antagonistic pattern between pathogen and host cells. To summarize, our findings showed that SARS-CoV-2 induced the significantly high expression of the cellular serine protease TMPRSS2 at 0 hpi to help the entry of viral particles into cells (Hoffmann et al., 2020; Figure 4). At the same time, host cell initiated an immediate response for the invasion of SARS-CoV-2 virus (Figure 1D). Then, the virus successfully suppressed the acute response of host cells for fast proliferation by increasing the transcripts of its 3′ genome end, including M, 6, 7a, 7b, 8, and N genes, which were consistent with their reported regulations to host immune response (Lim et al., 2016; Fung and Liu, 2019). As a response from host cell, a number of antiviral pathways and cytokine productions were up-regulated to resist the virus infection (Figures 2, 3). In particular, several metabolism-associated pathways were down-regulated at 12 and 24 hpi (Figure 2). After the antagonistic cycle, a dramatic proliferation of viral genome or transcriptome RNA was detected in the early infection of host cells (Figure 1B), which could possibly be an explanation for the fast spread of SARS-CoV-2 in humans. In addition to suppression of host responses by viral proteins, there was a possibility that the replication speed of SARS-CoV-2 could lead to the distinct host cellular response. However, simultaneous detection of SARS-CoV-2 viruses in cell culture supernatants at indicated infection time points showed that the replication level of SARS-CoV-2 (Supplementary Figure S4) seemed similar to those of SARS-CoV and MERS-CoV (Josset et al., 2013).

As SARS-CoV-2 reported a relatively low risk of mortality (Verity et al., 2020) compared with the other two serious human coronaviruses, SARS-CoV and MERS-CoV, we compared and contrasted the host transcriptomes in response to the viral infections. We found that some cytokines in SARS-CoV-2-infected cells were markedly up-regulated at a very early stage, which was not observed for SARS-CoV and MERS-CoV and even less frequently observed for other viruses. The unusual high expression of cytokines at 0 hpi possibly explains why patients with severe clinical symptoms rapidly deteriorated. Although the number of infected cases was very high, the majority of infections displayed mild symptoms that are partly explained by a gradual increase in host antiviral capability from 7 to 24 hpi. In contrast to SARS-CoV-2, both SARS-CoV and MERS-CoV were able to inhibit the antiviral capability of the host significantly, which could explain their observed relatively high mortalities. MERS was associated with a higher mortality than SARS, which could be in part attributed to the higher expression of cytokines suppressing the antiviral responses.

Recently, Blanco-Melo et al. (2020) have published transcriptome data of host responses to SARS-CoV-2 from *in vitro* cell lines including A549 (MOI of 0.2) and NHBE (MOI of 2) at 24 hpi. This previously published data is complemented by our study designed to investigate the early response phase of cell lines infected with SARS-CoV-2. While the previous work did not observe the elevated levels of IFNB1, IFNL1, and IFNL3, our findings show that not only IFNB1 but also IFNL1 and IFNL3 expressions are up-regulated between 7 and 24 hpi (Figure 3D). Also, they did not detect the gene expression of ACE2 and TMPRSS2 at 24 hpi, whereas we observed that ACE2 is down-regulated at 24 hpi and TMPRSS2 is only up-regulated at 0 hpi before returning to the normal levels (Figure 4). Our time-series sampling revealed distinct early response features of SARS-CoV-2, which provided a possible explanation for some clinical observations. For example, a recent clinical study (Wolfel et al., 2020) found that SARS-CoV-2 could replicate effectively in upper respiratory tract tissues, and that the viral loads appeared earlier (before day 5) and were substantially more than expected. Findings from the present study have confirmed that, at 7 hpi, the 3′ end of SARS-CoV-2 genome starts to express densely, reducing the effectiveness of host immune surveillance, which possibly enables the rapid replication of SARS-CoV-2 in upper respiratory tract tissues.

In spite of the fact that several studies have already demonstrated a consistent correlation between gene expression measured by RNA-Seq and by microarray (Nookaew et al., 2012; Guo et al., 2013; Chen et al., 2017), we still need to exclude the possibility of bias resulting from different methodologies. First, because RNA-Seq can potentially detect more genes than microarrays, we only considered protein-coding genes for the analysis of RNA-Seq results. For SARS-CoV-2, the microarray analysis identified more than 90% of the 6,794 DEGs, including 6,514 DEGs of SARS-CoV and 6,198 DEGs of MERS-CoV. Second, expressions of the 6,800 DEGs were distributed over the four time points from low to high, not only in SARS-CoV-2 but also in SARS-CoV and MERS-CoV (Supplementary Figures S5, S6), indicating that the silent early host responses to SARS-CoV and MERS-CoV appeared not to be due to technological biases. Lastly, when extending the infection time from 24 to 72 hpi (GSE33267), thousands of DEGs (minimum 1,022 and maximum 2,017 genes), which had been inhibited at the early stages, were actually induced (Supplementary Figure S7).

During SARS-CoV-2 infection, reads mapped to host genomes actually decreased, which possibly biased gene expression quantification. However, enough host reads were still obtained for the infected samples (Supplementary Table S1), that is, an average of 22,429,835 (infected 0 h) vs. 20,327,755 (mock 0 hpi), 18,175,274 (infected 7 hpi) vs. 22,821,495 (mock 7 hpi), 5,272,219 (infected 12 hpi) vs. 21,575,326 (mock 12 hpi), and 5,130,244 (infected 24 hpi) vs. 18,567,346 (mock 24 hpi). Notably, in the early stages (0 and 7 hpi), about 20 M reads were sufficient for accurate quantification of gene expression level, which can solidly support our main findings in the early stages of infection. Furthermore, qRT-PCR experiments showed consistent gene expression levels between RNA-Seq and qRT-PCR technologies (Figure 5).

With respect to Calu-3 cells, the two public datasets used a sub-population of Calu-3 cells (Calu-3 2B4) that were sorted by ACE2 antibody in order to help virus entry into host cells, whereas our dataset used the mixed Calu-3 cells with low and high ACE2 expressions. To allow similar cell entry, thus, a little longer incubation time (2 h) was taken for SARS-CoV-2 than those for SARS-CoV and MERS-CoV (40 min) (Josset et al., 2013). Although MERS-CoV used a different receptor, DPP4, comparisons among three viruses in Calu-3 cells were still reasonable for two reasons. Firstly, DPP4 had relatively similar expression levels to ACE2 in Calu-3 cells (Supplementary Figure S8). Secondly, SARS-CoV and MERS-CoV had very similar replication kinetics within 24 h (Josset et al., 2013).

## Data Availability Statement

The datasets presented in this study can be found in online repositories. The names of the repository/repositories and accession number(s) can be found below: National Genomics Data Center (https://bigd.big.ac.cn/) with the accession number PRJCA002617. https://bigd.big.ac.cn/bioproject/browse/PRJCA002617.

## Author Contributions

TJ, WT, BH, and ZX devised the experiment and wrote the manuscript. JSu, AW, JSh, and WZ conducted bioinformatics analysis. FY, BH, and RY prepared the samples. MW provided the Calu-3 cell. MP did the RNA sequencing and qRT-PCR. All authors revised the manuscript.

## Conflict of Interest

The authors declare that the research was conducted in the absence of any commercial or financial relationships that could be construed as a potential conflict of interest.

## Footnotes

1 https://ftp.ncbi.nlm.nih.gov/gene/DATA/gene2go.gz

2 http://purl.obolibrary.org/obo/go/go-basic.obo

## Supplementary Material

Supplementary Figure 1 Volcano plot of gene expression levels during SARS-CoV-2 infection. The red color represents up-regulated DEGs, and the blue color represents down-regulated DEGs. The arrows point to DEG numbers corresponding to different cutoffs of log2FC.

Supplementary Figure 2 Variation of the number of up-regulated genes minus down-regulated genes over time.

Supplementary Figure 3 GO enrichment analysis of DEG patterns for SARS-CoV-2. The numbers in parentheses indicate gene numbers of DEG patterns. The heatmap color represents −log10 (enrichment *P*-value).

Supplementary Figure 4 SARS-CoV-2 replication in Calu-3 cells. SARS-CoV-2 titers in cell culture supernatants at indicated infection time points (*n* = 3 independent biological replicates). The error bars represent mean ± SD.

Supplementary Figure 5 Expression levels of cytokine genes between the mock and infected groups. The left column is for SARS-CoV-2, the middle column for SARS-CoV, and the right column for MERS-CoV. For SARS-CoV-2, the expression level is quantified by log2(TPM + 1). Cytokine genes are highlighted.

Supplementary Figure 6 Expression levels of antivirus-related genes between the mock and infected groups. The left, middle, and right columns show results for SARS-CoV-2, SARS-CoV, and MERS-CoV, respectively. For SARS-CoV-2, the expression level is quantified by log2(TPM + 1). Antiviral-related genes are highlighted.

Supplementary Figure 7 Variation of the number of DEGs during SARS-CoV infection.

Supplementary Figure 8 Gene expression levels of DPP4 and ACE2 in mock samples for three viruses. Each time point includes three biological mock samples.

## References

[B1] Anders S. Pyl P. T. Huber W. (2015). HTSeq–a Python framework to work with high-throughput sequencing data. *Bioinformatics* 31 166–169. 10.1093/bioinformatics/btu638 25260700 PMC4287950

[B2] Blanco-Melo D. Nilsson-Payant B. E. Liu W.-C. Møller R. Panis M. Sachs D. (2020). SARS-CoV-2 launches a unique transcriptional signature from in vitro, ex vivo, and in vivo systems. *bioRxiv* 10.1101/2020.03.24.004655

[B3] Bolger A. M. Lohse M. Usadel B. (2014). Trimmomatic: a flexible trimmer for illumina sequence data. *Bioinformatics* 30 2114–2120. 10.1093/bioinformatics/btu170 24695404 PMC4103590

[B4] Carrasco Pro S. Dafonte Imedio A. Santoso C. S. Gan K. A. Sewell J. A. Martinez M. (2018). Global landscape of mouse and human cytokine transcriptional regulation. *Nucleic Acids Res.* 46 9321–9337. 10.1093/nar/gky787 30184180 PMC6182173

[B5] Channappanavar R. Perlman S. (2017). Pathogenic human coronavirus infections: causes and consequences of cytokine storm and immunopathology. *Semin. Immunopathol.* 39 529–539. 10.1007/s00281-017-0629-x 28466096 PMC7079893

[B6] Chen L. Sun F. Yang X. Jin Y. Shi M. Wang L. (2017). Correlation between RNA-Seq and microarrays results using TCGA data. *Gene* 628 200–204. 10.1016/j.gene.2017.07.056 28734892

[B7] Daugherty M. D. Young J. M. Kerns J. A. Malik H. S. (2014). Rapid evolution of PARP genes suggests a broad role for ADP-ribosylation in host-virus conflicts. *PLoS Genet.* 10:e1004403. 10.1371/journal.pgen.1004403 24875882 PMC4038475

[B8] Dobin A. Davis C. A. Schlesinger F. Drenkow J. Zaleski C. Jha S. (2013). STAR: ultrafast universal RNA-seq aligner. *Bioinformatics* 29 15–21. 10.1093/bioinformatics/bts635 23104886 PMC3530905

[B9] Eisenberg E. Levanon E. Y. (2013). Human housekeeping genes, revisited. *Trends Genet.* 29 569–574. 10.1016/j.tig.2013.05.010 23810203

[B10] El-Diwany R. Soliman M. Sugawara S. Breitwieser F. Skaist A. Coggiano C. (2018). CMPK2 and BCL-G are associated with type 1 interferon-induced HIV restriction in humans. *Sci. Adv.* 4:eaat0843. 10.1126/sciadv.aat0843 30083606 PMC6070316

[B11] Fung T. S. Liu D. X. (2019). Human coronavirus: host-pathogen interaction. *Annu. Rev. Microbiol.* 73 529–557. 10.1146/annurev-micro-020518-115759 31226023

[B12] Guan W. J. Ni Z. Y. Hu Y. Liang W. H. Ou C. Q. He J. X. (2020). Clinical characteristics of coronavirus disease 2019 in China. *N. Engl. J. Med.* 382 1708–1720. 32109013 10.1056/NEJMoa2002032 PMC7092819

[B13] Guo Y. Sheng Q. Li J. Ye F. Samuels D. C. Shyr Y. (2013). Large scale comparison of gene expression levels by microarrays and RNAseq using TCGA data. *PLoS One* 8:e71462. 10.1371/journal.pone.0071462 23977046 PMC3748065

[B14] Hoffmann M. Kleine-Weber H. Schroeder S. Kruger N. Herrler T. Erichsen S. (2020). SARS-CoV-2 cell entry depends on ACE2 and TMPRSS2 and is blocked by a clinically proven protease inhibitor. *Cell* 181 271.e–280.e. 32142651 10.1016/j.cell.2020.02.052 PMC7102627

[B15] Huang C. Wang Y. Li X. Ren L. Zhao J. Hu Y. (2020). Clinical features of patients infected with 2019 novel coronavirus in Wuhan, China. *Lancet* 395 497–506. 31986264 10.1016/S0140-6736(20)30183-5 PMC7159299

[B16] Josset L. Menachery V. D. Gralinski L. E. Agnihothram S. Sova P. Carter V. S. (2013). Cell host response to infection with novel human coronavirus EMC predicts potential antivirals and important differences with SARS coronavirus. *mBio* 4:e00165-13. 10.1128/mBio.00165-13 PMC3663187 23631916

[B17] Kell A. M. Gale M. Jr. (2015). RIG-I in RNA virus recognition. *Virology* 47 110–121. 10.1016/j.virol.2015.02.017 25749629 PMC4424084

[B18] Lazear H. M. Lancaster A. Wilkins C. Suthar M. S. Huang A. Vick S. C. (2013). IRF-3, IRF-5, and IRF-7 coordinately regulate the type I IFN response in myeloid dendritic cells downstream of MAVS signaling. *PLoS Pathog.* 9:e1003118. 10.1371/journal.ppat.1003118 23300459 PMC3536698

[B19] Li Q. Guan X. Wu P. Wang X. Zhou L. Tong Y. (2020). Early transmission dynamics in Wuhan, China, of novel coronavirus-infected pneumonia. *N. Engl. J. Med.* 382 1199–1207. 31995857 10.1056/NEJMoa2001316 PMC7121484

[B20] Li W. Moore M. J. Vasilieva N. Sui J. Wong S. K. Berne M. A. (2003). Angiotensin-converting enzyme 2 is a functional receptor for the SARS coronavirus. *Nature* 426 450–454. 14647384 10.1038/nature02145 PMC7095016

[B21] Lim Y. X. Ng Y. L. Tam J. P. Liu D. X. (2016). Human coronaviruses: a review of virus-host interactions. *Diseases* 4:26. 10.3390/diseases4030026 28933406 PMC5456285

[B22] Love M. I. Huber W. Anders S. (2014). Moderated estimation of fold change and dispersion for RNA-seq data with DESeq2. *Genome Biol.* 15:550. 10.1186/s13059-014-0550-8 PMC4302049 25516281

[B23] Nookaew I. Papini M. Pornputtapong N. Scalcinati G. Fagerberg L. Uhlen M. (2012). A comprehensive comparison of RNA-Seq-based transcriptome analysis from reads to differential gene expression and cross-comparison with microarrays: a case study in Saccharomyces cerevisiae. *Nucleic Acids Res.* 40 10084–10097. 10.1093/nar/gks804 22965124 PMC3488244

[B24] Perez-Caballero D. Zang T. Ebrahimi A. McNatt M. W. Gregory D. A. Johnson M. C. (2009). Tetherin inhibits HIV-1 release by directly tethering virions to cells. *Cell* 139 499–511. 10.1016/j.cell.2009.08.039 19879838 PMC2844890

[B25] Qin C. Zhou L. Hu Z. Zhang S. Yang S. Tao Y. (2020). Dysregulation of immune response in patients with COVID-19 in Wuhan, China. *Clin. Infect. Dis.* 71 762–768. 10.1093/cid/ciaa248 32161940 PMC7108125

[B26] Raj V. S. Mou H. Smits S. L. Dekkers D. H. Muller M. A. Dijkman R. (2013). Dipeptidyl peptidase 4 is a functional receptor for the emerging human coronavirus-EMC. *Nature* 495 251–254. 10.1038/nature12005 23486063 PMC7095326

[B27] Ritchie M. E. Phipson B. Wu D. Hu Y. Law C. W. Shi W. (2015). limma powers differential expression analyses for RNA-sequencing and microarray studies. *Nucleic Acids Res.* 43:e47. 10.1093/nar/gkv007 25605792 PMC4402510

[B28] Schreiber G. Piehler J. (2015). The molecular basis for functional plasticity in type I interferon signaling. *Trends Immunol.* 36 139–149. 10.1016/j.it.2015.01.002 25687684

[B29] Sims A. C. Tilton S. C. Menachery V. D. Gralinski L. E. Schafer A. Matzke M. M. (2013). Release of severe acute respiratory syndrome coronavirus nuclear import block enhances host transcription in human lung cells. *J. Virol.* 87 3885–3902. 10.1128/jvi.02520-12 23365422 PMC3624188

[B30] Song Z. Xu Y. Bao L. Zhang L. Yu P. Qu Y. (2019). From SARS to MERS, thrusting coronaviruses into the spotlight. *Viruses* 11:59. 10.3390/v11010059 30646565 PMC6357155

[B31] Sun J. Wang J. Yuan X. Wu X. Sui T. Wu A. (2019). Regulation of early host immune responses shapes the pathogenicity of avian Influenza A virus. *Front. Microbiol.* 10:2007. 10.3389/fmicb.2019.02007 31572308 PMC6749051

[B32] Sun J. Ye F. Wu A. Yang R. Pan M. Sheng J. (2020). Comparative transcriptome analysis reveals the intensive early-stage responses of host cells to SARS-CoV-2 infection. *bioRxiv* 10.1101/2020.04.30.071274 PMC7723856 33324374

[B33] Totura A. L. Whitmore A. Agnihothram S. Schafer A. Katze M. G. Heise M. T. (2015). Toll-like receptor 3 signaling via TRIF contributes to a protective innate immune response to severe acute respiratory syndrome coronavirus infection. *mBio* 6:e00638-15. 10.1128/mBio.00638-15 PMC4447251 26015500

[B34] Verity R. Okell L. C. Dorigatti I. Winskill P. Whittaker C. Imai N. (2020). Estimates of the severity of coronavirus disease 2019: a model-based analysis. *Lancet Infect. Dis.* 20 669–677. 32240634 10.1016/S1473-3099(20)30243-7 PMC7158570

[B35] WHO (2020). *Coronavirus Disease (COVID-19).* Geneva: WHO.

[B36] Wolfel R. Corman V. M. Guggemos W. Seilmaier M. Zange S. Muller M. A. (2020). Virological assessment of hospitalized patients with COVID-2019. *Nature* 581 465–469. 10.1038/s41586-020-2196-x 32235945

[B37] Wu A. Peng Y. Huang B. Ding X. Wang X. Niu P. (2020). Genome composition and divergence of the novel coronavirus (2019-nCoV) originating in China. *Cell Host Microbe* 27 325–328. 10.1016/j.chom.2020.02.001 32035028 PMC7154514

[B38] Xiong Y. Liu Y. Cao L. Wang D. Guo M. Jiang A. (2020). Transcriptomic characteristics of bronchoalveolar lavage fluid and peripheral blood mononuclear cells in COVID-19 patients. *Emerg. Microbes Infect.* 9 761–770. 10.1080/22221751.2020.1747363 32228226 PMC7170362

[B39] Xu Z. Shi L. Wang Y. Zhang J. Huang L. Zhang C. (2020). Pathological findings of COVID-19 associated with acute respiratory distress syndrome. *Lancet Respir. Med.* 8 420–422. 10.1016/s2213-2600(20)30076-x 32085846 PMC7164771

[B40] Yu M. Zhang C. Yang Y. Yang Z. Zhao L. Xu L. (2011). The interaction between the PARP10 protein and the NS1 protein of H5N1 AIV and its effect on virus replication. *Virol. J.* 8:546. 10.1186/1743-422x-8-546 22176891 PMC3287249

[B41] Zhu N. Zhang D. Wang W. Li X. Yang B. Song J. (2020). A novel coronavirus from patients with pneumonia in China, 2019. *N. Engl. J. Med.* 382 727–733. 31978945 10.1056/NEJMoa2001017 PMC7092803

