# Supplementary figures and images for: Comparative Transcriptome Analysis Reveals the Intensive Early Stage Responses of Host Cells to SARS-CoV-2 Infection
Source: Front Microbiol. 2020 Nov 25;11:593857. doi: 10.3389/fmicb.2020.593857 (PMC7723856; doi:10.3389/fmicb.2020.593857)

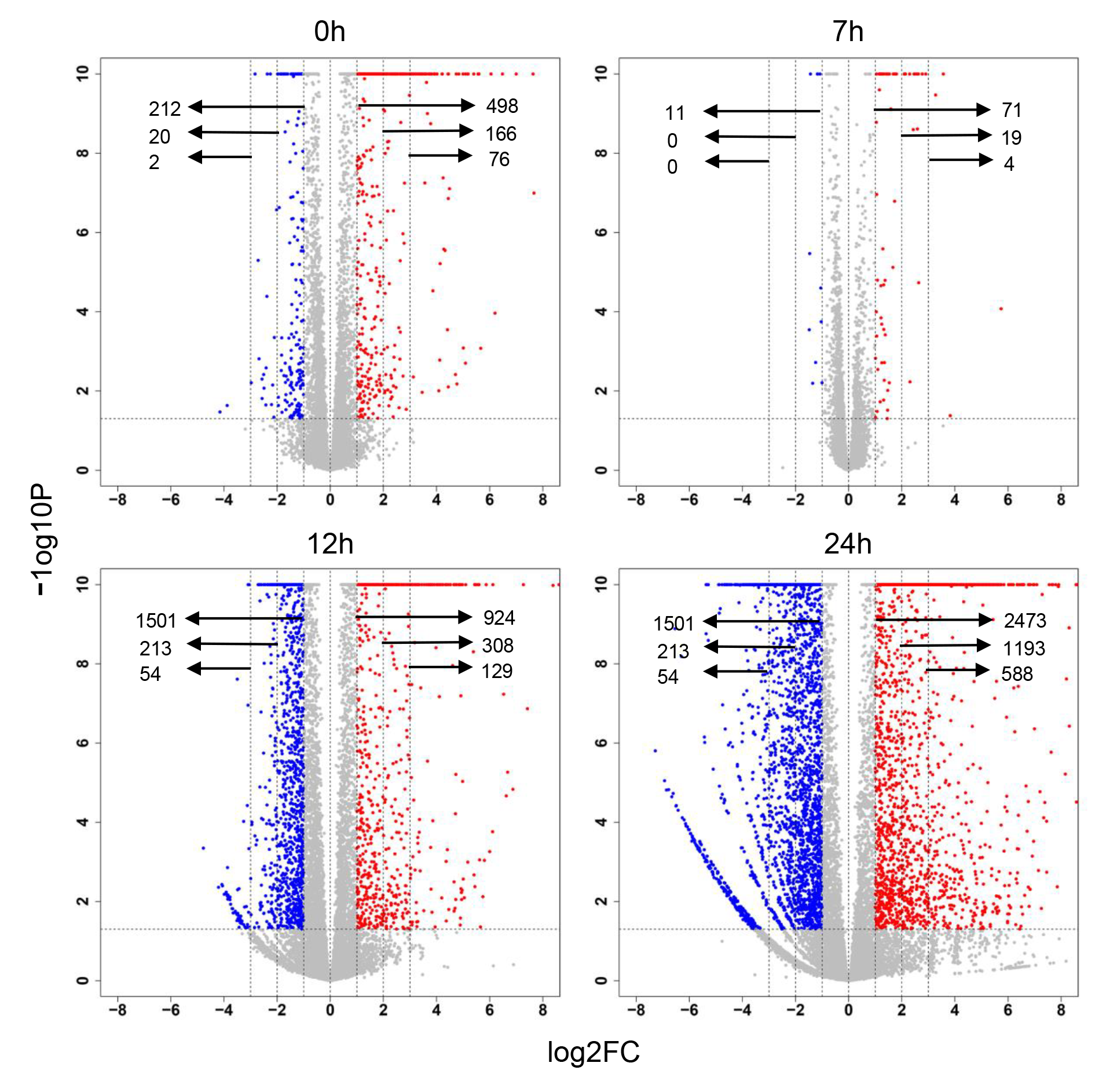

Supplement: Supplementary Figure 1 — Volcano plot of gene expression levels during SARS-CoV-2 infection. The red color represents up-regulated DEGs, and the blue color represents down-regulated DEGs. The arrows point to DEG numbers corresponding to different cutoffs of log2FC. [file Image_1.TIF]

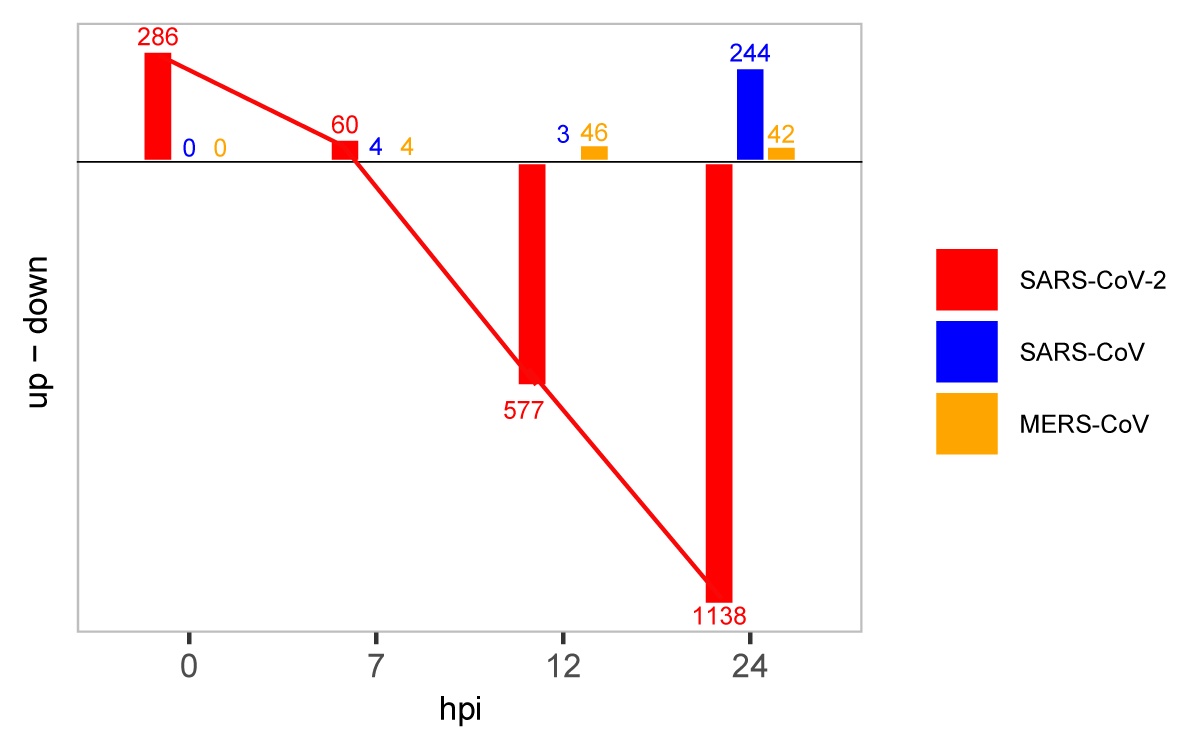

Supplement: Supplementary Figure 2 — Variation of the number of up-regulated genes minus down-regulated genes over time. [file Image_2.TIF]

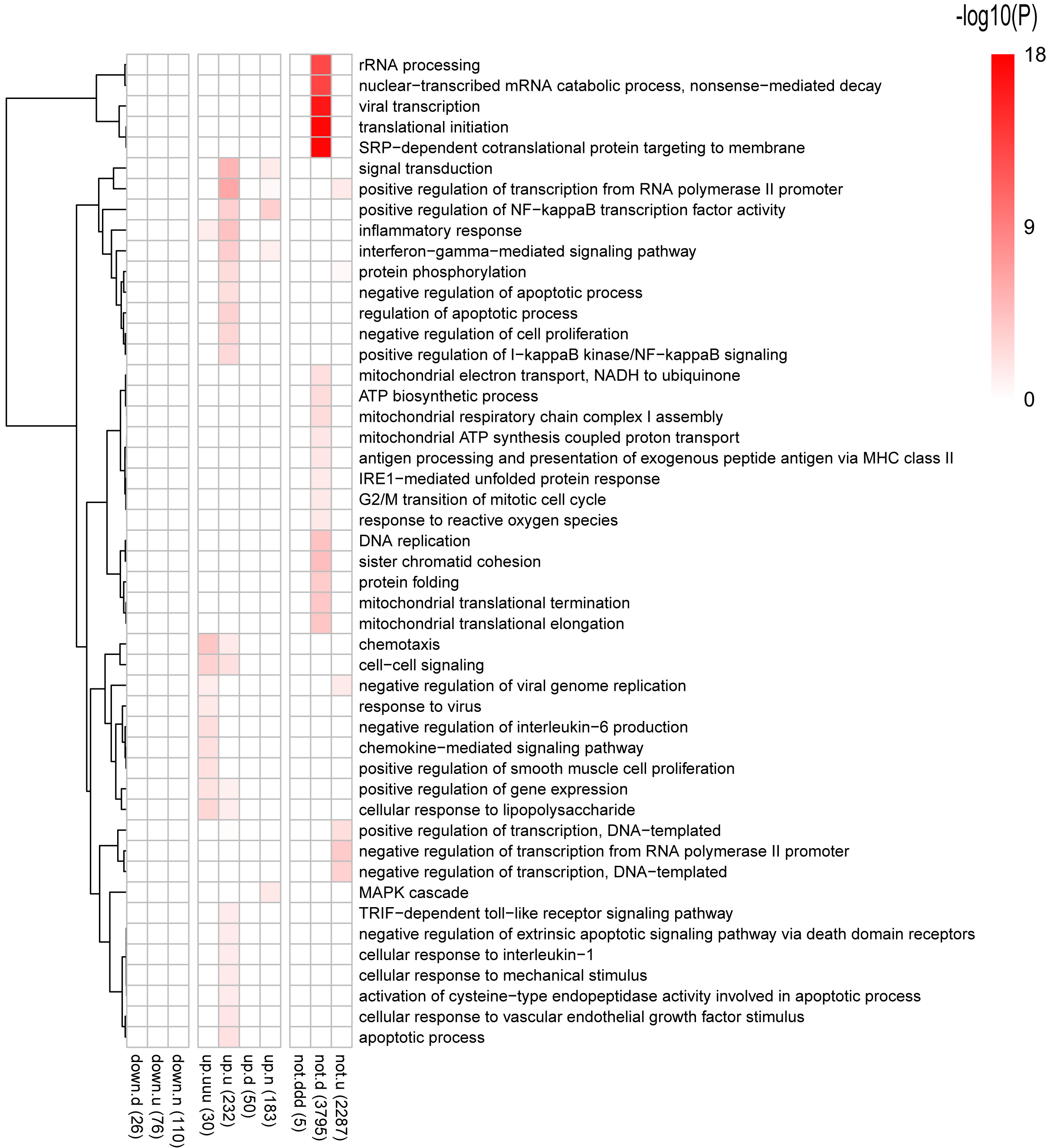

Supplement: Supplementary Figure 3 — GO enrichment analysis of DEG patterns for SARS-CoV-2. The numbers in parentheses indicate gene numbers of DEG patterns. The heatmap color represents −log10 (enrichment P-value). [file Image_3.TIF]

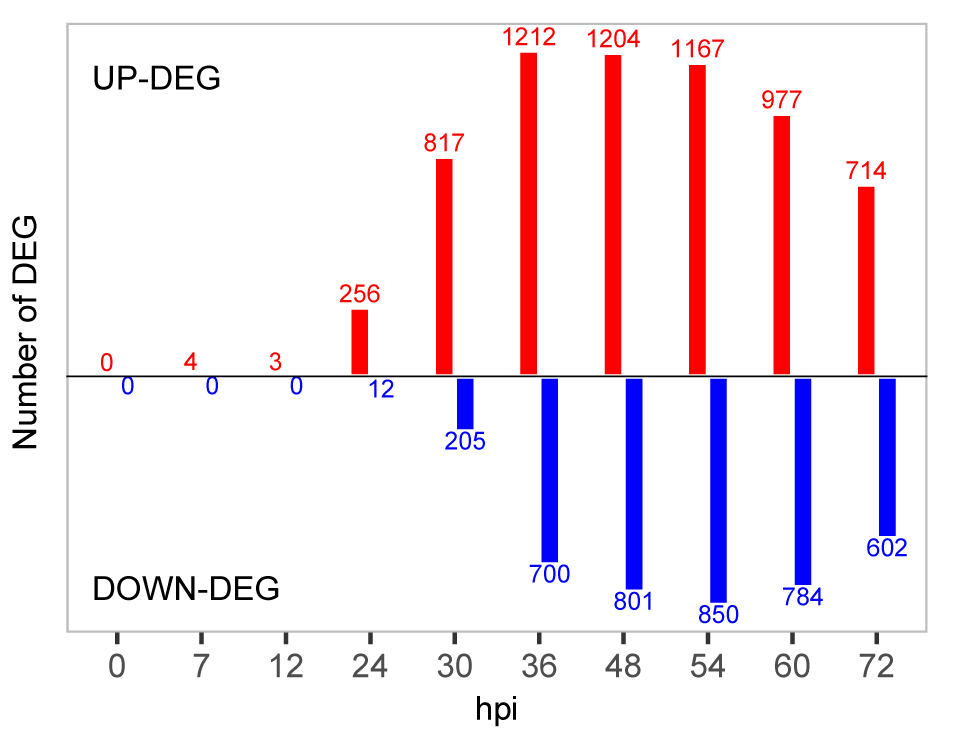

Supplement: Supplementary Figure 7 — Variation of the number of DEGs during SARS-CoV infection. [file Image_7.TIF]

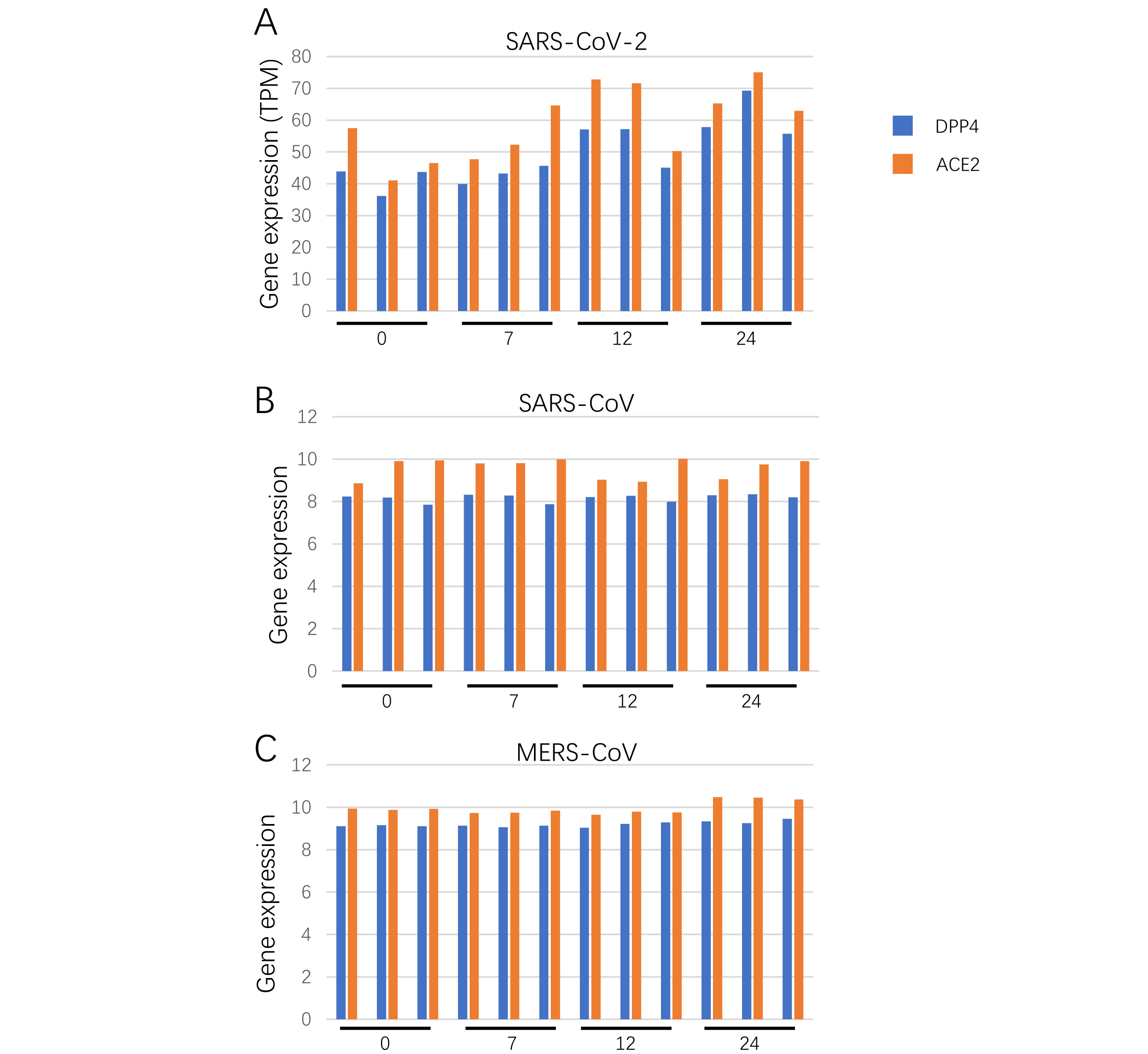

Supplement: Supplementary Figure 8 — Gene expression levels of DPP4 and ACE2 in mock samples for three viruses. Each time point includes three biological mock samples. [file Image_8.TIF]
